# LncRNA MALAT1 functions as a biomarker of no-reflow phenomenon in ST-segment elevation myocardial infarction patients receiving primary percutaneous coronary intervention

**DOI:** 10.1038/s41598-022-06923-z

**Published:** 2022-02-28

**Authors:** Xiheng Yang, Rixin Dai, Zhong Qin, Ruping Cai, Yuli Xu, Qiang Su

**Affiliations:** Department of Cardiology, Affiliated Hospital of Guilin Medical University, 15#, Lequn Road, Guilin, 541001 Guangxi China

**Keywords:** Molecular biology, Biomarkers

## Abstract

MALAT1 was reported to sponge miR-30e, miR-126 and miR-155 in the pathogenesis of many diseases. Plasma miR-30e can indicate the risk of no-reflow during primary percutaneous coronary intervention (pPCI), while miR-126 can be used as a predictor of coronary slow flow phenomenon. In this study, we compared the diagnostic value of above genes in the prediction of no-reflow phenomenon in ST-segment elevation myocardial infarction (STEMI) subjects receiving pPCI. Quantitative real-time PCR, ELISA, Western blot and luciferase assays were performed to explore the regulatory relationship of MALAT1/miR-30e, MALAT1/miR-126, MALAT1/miR-155, miR-126/HPSE, and miR-155/EDN1. ROC analysis was carried out to evaluate the potential value of MALAT1, miRNAs and target genes in differentiating normal reflow and no-reflow in STEMI patients receiving pPCI. Elevated MALAT1, CRP, HPSE, and EDN1 expression and suppressed miR-30e, miR-155 and miR-126 expression was found in the plasma of STEMI patients receiving pPCI who were diagnosed with no-reflow phenomenon. ROC analysis showed that the expression of MALAT1, miR-30e, miR-126 and CRP could be used as predictive biomarkers to differentiate normal reflow and no-reflow in STEMI patients receiving pPCI. MALAT1 was found to suppress the expression of miR-30e, miR-126 and miR-155, and HPSE and EDN1 were respectively targeted by miR-126 and miR-155. This study demonstrated that MALAT1 could respectively sponge the expression of miR-30e, miR-126 and miR-155. And miR-30e, miR-126 and miR-155 respectively targeted CRP, HPSE and EDN1 negatively. Moreover, MALAT1 could function as an effective biomarker of no-reflow phenomenon in STEMI patients receiving pPCI.

## Introduction

No-reflow is defined by insufficient myocardial perfusion despite mechanical opening of the corresponding lesion via percutaneous coronary intervention (PCI)^1^. No-reflow occurs in about 10% of primary PCI patients^2^. Also, microvascular obstructions as a result of distal embolization, thrombosis, as well as microvascular contraction have been suggested as mechanisms underlying no-reflow, which are associated with many adverse results such as stroke, heart failure, as well as cardiac mortality^3,4^. Moreover, parameters such as CHA2DS2-VASc score, which estimates the risk of thromboembolism in patients with atrial fibrillation, can also predict no-reflow in patients who underwent pPCI^5^. Although many factors have been suggested as risk factors of no-reflow, no widely approved procedure is available to stratify the risk of no-reflow.

Long non-coding RNAs (lncRNAs) are transcripts containing ≥ 200 nucleotides. In recent studies, lncRNAs have attracted great recognition due to their important role in many biological processes such as the differentiation, growth, homeostasis, as well as embryonic development of cells^6,7^. Metastasis-associated lung adenocarcinoma transcript 1 (MALAT1) is a conserved lncRNA implicated in lung metastasis and poor prognosis of lung cancer patients^8,9^. Latest studies revealed that MALAT1 exerted an essential impact on the regulation of many pathophysiological processes such as neurologic disorders, vascular diseases, and cancers by affecting the proliferation, migration, apoptosis and invasion of cancer cells^10^.

Just recently, the elevated pre-operative value of C-reactive protein (CRP) was shown to predict the prognosis of survival in pNEN patients^11^. It was also shown that CRP caused upregulated COX-1 and COX-2 expression to reduce inflammation^12^. Some studies disclosed that COX-2 exerted an essential effect on the angiogenesis induced by IL-1b^13,14^. Heparanase (HPSE) was an enzyme recently found to play an important role to degrade the heparan sulfate (HS) chains in glycosaminoglycans. As a biologically active proteoglycan, HS is implicated in many important processes of adhesion reactions by interacting with cytokines, adhesion molecules, and signaling molecules in cells, thus impacting cell differentiation, proliferation and migration. HPSE can also promote the invasion, metastasis, chemotaxis and division of tumor cells^15,16^. The levels of HPA were additionally shown to be higher in PCI patients with the no-reflow phenomenon^17^. Also, elevated levels of HPA in STEMI patients were linked to a higher incidence of TB. On top of that, raised HPA levels may be linked to thrombotic complications including no-reflow in STEMI patients. Endothelin-1 (ET-1) is an effective vasoconstrictor, mitogen, and pro-inflammatory mediator generated upon vessel wall stress and hypoxia. ET-1 also plays an important role in endothelial inflammation and the formation of atherosclerotic plaques^18^. Additionally, the network structure of stents can aggravate microcirculation dysfunction to induce chemotaxis and trigger monocyte activation to secrete various vasoactive factors like ET-1 and VCAM-1, which in turn induce the onset of reperfusion injury^19,20^.

MALAT1 has been reported to regulate the miR-155 expression and promote the proliferation and migration of cardiac stem cells under hypoxia^21^. Apart from miR-155, many miRNAs including miR-30e and miR-126 were proved to be ‘sponged’ by MALAT1 in the pathogenesis of many diseases^22,23^. Moreover, these miRNAs have been reported to function as biomarkers in STEMI patients. Plasma miR-30e could indicate the risk of no-reflow during pPCI, while miR-126 could be used as a predictor of coronary slow flow phenomenon^24^. Furthermore, several proteins were also implicated in cardiac diseases. For example, increased HPSE expression was shown to be associated with no-reflow phenomenon in STEMI patients, and elevated EDN-1 indicated increased coronary instability^17,25^. In this study, we compared the diagnostic value of the above genes in the prediction of no-reflow phenomenon in STEMI subjects receiving primary PCI.

## Materials and methods

### Human subjects and sample collection

In this study, we recruited 198 ST-segment elevation myocardial infarction (STEMI) patients receiving primary percutaneous coronary intervention (pPCI) during August 2018 to May 2021 in the department of cardiology at the Affiliated Hospital of Guilin Medical University. The patients were continuously enrolled and divided into two groups according to their reflow status as the normal reflow group (n = 152) and the no-reflow group (N = 46). No-reflow is diagnosed as the persistence of forward blood flow disorder [TIMI flow grade ≤ 2] after coronary artery without mechanical obstruction and no significant residual stenosis or dissection. The age, gender, diabetes history, hypertension history, hyperlipidemia history, familial history, smoking history, chest pain to hospitalization time, and door to balloon time, the angiography and procedural characteristics including infarct-related artery, multi-vessel disease, stent implantation and total stent length were collected and summarized for subsequent comparison. And serum samples were collected from all participants before their pPCI treatment. The ethical committee of Affiliated Hospital of Guilin Medical University has approved the protocol of this study (Approval ID: 2018-08-012). All procedures were performed in strict accordance with the last vision of the Declaration of Helsinki. Written informed consent was obtained from all patients or their first-degree relatives before the study.

### RNA isolation and real-time PCR

In this study, real-time PCR was done to compare the expression of MALAT1, miR-30e, miR-126, miR-155, CRP mRNA, HPSE mRNA, and EDN1 mRNA in each sample. In brief, total RNA in each sample was separated by utilizing a Trizol ® reagent (Invitrogen, Carlsbad, CA) following the standard assay procedure provided on the instruction manual of the supplier. In the next step, the quality and content of separated RNA was quantified at 260 nm and 280 nm absorbance by using a Nanodrop ND-3000 device (Thermo Fisher Scientific, Waltham, MA) following the standard assay procedure provided on the instruction manual of the instrument supplier. Then, 300 ng of separated total RNA from each sample were used to synthesis cDNA templates using a cDNA reverse transcription assay kit (Thermo Fisher Scientific, Waltham, MA) following the standard assay procedure provided on the instruction manual of the assay kit supplier. Finally, a Taqman Gene Expression Assay kit (Invitrogen, Carlsbad, CA) was used following the standard assay procedure provided on the instruction manual of the assay kit supplier to determine the relative expression of MALAT1, miR-30e, miR-126, miR-155, CRP mRNA, HPSE mRNA, and EDN1 mRNA in each sample via real time qPCR, which was carried out on a StepOnePlus (Invitrogen, Carlsbad, CA) real time qPCR machine following the standard assay procedure provided on the instruction manual of the instrument manufacturer. The relative expression of MALAT1, miR-30e, miR-126, miR-155, CRP mRNA, HPSE mRNA, and EDN1 mRNA in each sample was calculated using the Ct method and then standardized to the expression level of internal control β-actin.

### Cell culture and transfection

HUVEC and HAEC cells were bought from Lonza (Walkersville, MD) and maintained following the standard incubation procedure provided by the supplier. In brief, the cells were maintained at 37 °C and 5% CO_2_ in DMEM media (Gibco, Thermo Fisher Scientific, Waltham, MA) added with 10% FBS and penstrep. When HUVEC and HAEC cells reached confluency, they were divided into two groups, i.e., 1. NC siRNA group (HUVEC and HAEC cells transfected with negative control siRNA); and 2. MALAT1 siRNA group (HUVEC and HAEC cells transfected with MALAT1 siRNA). The transfection was carried out using Lipofectamine 3000 (Invitrogen, Carlsbad, CA) following the standard transfection procedure provided on the instruction manual of the transfection reagent supplier, and transfected cells were collected 48 h later to assay the expression of target genes.

### Vector construction, mutagenesis, and luciferase assay

Our binding site screening results showed that miR-30e could potentially bind to MALAT1, so the luciferase vectors containing wild type and mutant promoter of MALAT1 carrying the miR-30e binding site were established and transfected into HUVEC and HAEC cells with miR-30e. In brief, the wild-type promoter sequence of MALAT1 carrying the miR-30e binding site was inserted into a pcDNA vector (Promega, Madison, WI) downstream the luciferase reporter gene to generate the wild type plasmid for the MALAT1. Then, a Quick Change III mutagenesis kit (Stratagene, San Diego, CA) was used following the standard assay procedure provided on the instruction manual of the assay kit supplier to induce a site-directed mutation in the miR-30e binding site of MALAT1 promoter, and the mutant sequence was also inserted into a pcDNA vector downstream the luciferase reporter gene to generate the mutant type plasmid for the MALAT1 promoter. In the next step, both luciferase vectors containing wild type and mutant MALAT1 were co-transfected into HUVEC and HAEC cells along with miR-30e, and the luciferase activity of transfected HUVEC and HAEC cells was assayed 48 h later using a Dual-Luciferase Report gene assay kit (Promega, Madison, WI) following the standard assay procedure provided on the instruction manual of the assay kit. Similarly, our binding site screening results showed that miR-126 could potentially bind to MALAT1, so the luciferase vectors containing wild type and mutant promoter of MALAT1 carrying the miR-126 binding site were established and transfected into HUVEC and HAEC cells with miR-126, according to the method similar to the one described above. In addition, to study the regulatory relationship of MALAT1/miR-155, miR-126/HPSE, and miR-155/EDN1, luciferase vectors containing wild type and mutant MALAT1, HPSE or EDN1 were established and co-transfected into HUVEC and HAEC cells along with miR-155, miR-126 and miR-155, respectively, and the luciferase activity of transfected HUVEC and HAEC cells was assayed 48 h later using the Dual-Luciferase Report gene assay kit. The reading of luciferase activity of transfected HUVEC and HAEC cells was done in a Turner luminometer (Turner Biosystems Luminometer, Promega, Madison, WI) following the standard assay procedure provided on the instruction manual of the luminometer.

### Western blot analysis

The total protein from each sample was isolated first by using a RIPA lysis buffer (Sangon Biotech, Shanghai, China) following the standard assay procedure provided on the instruction manual of the lysis buffer supplier. In the next step, the concentration of isolated protein was quantified by utilizing a bicinchoninic acid (BCA) protein assay kit (Thermo Fisher Scientific, Waltham, MA) following the standard assay procedure provided on the instruction manual of the assay kit supplier. Then, the total protein isolated from each sample was resolved on a 10% SDS-PAGE gel (Thermo Fisher Scientific, Waltham, MA) and blotted onto a PVDF membrane, which was then blotted in 5% skim milk and then incubated in sequence with primary anti-CRP, anti-HPSE, and anti-EDN1 antibodies as well as HRP-conjugated secondary antibodies (all antibodies were purchased from Santa Cruz biotechnology, Dallas, TX) following the standard incubation procedures provided on the instruction manual of the antibody supplier. Finally, after the PVDF membrane was developed in an enhanced chemiluminescence reagent (Thermo Fisher Scientific, Waltham, MA) following the standard assay procedure provided on the instruction manual of the reagent supplier, the protein bands were imaged and analyzed by using a chemiluminescence imaging system to calculate the relative protein expression of CRP, HPSE, and EDN1 in each sample.

### ELISA

The plasma concentrations of CRP, HPSE, and EDN1 proteins in each patient were determined by utilizing corresponding enzyme-linked immunosorbent assay kits (R&D systems, Minneapolis, MN) following the standard assay procedure provided on the instruction manual of the assay kit supplier.

### Statistical analysis

All statistical analyses were done by utilizing SPSS version 19 (SPSS, Chicago, IL) and Prism version 7.0 (GraphPad, San Diego, CA). Differences with P < 0.05 were deemed statistically significant differences in this study. The comparison of non-continuous data between two or more groups was evaluated by using Chi square test. The comparison of continuous data between two and more groups was evaluated by using student t-test and one-way ANOVA, respectively. The normal distribution of each group was evaluated by using Kolmogorov–Smirnov analysis. Inter-group comparisons were carried out using Student’s t-tests. The diagnostic value including both sensitivity and specificity was evaluated by using ROC analysis. The sample size was evaluated by using https://www.stat.ubc.ca/~rollin/stats/ssize/n2.html.

### Ethics approval and consent to participate

The ethical committee of Affiliated Hospital of Guilin Medical University has approved the protocol of this study (Approval ID: 2018-08-012).

## Results

### Multivariate logistic regression analysis of reflow-related features of STEMI patients

As shown in Table 1, no significant differences were observed in respect to parameters including age, gender, diabetes history, hypertension history, hyperlipidemia history, familial history, smoking history, chest pain to hospitalization time, and door to balloon time between the normal reflow and no-reflow patient groups. Moreover, no remarkable difference was observed in respect to the angiography and procedural characteristics including infarct related artery, multi-vessel disease, stent implantation and total stent length were compared between the two groups (Table 2). However, the expression of MALAT1, miR-30e, miR-126, CRP, HPSE and EDN1 showed apparent differences between the two groups (Table 3).

**Table 1:** Basic clinical features of STEMI patients.

Characteristics	Normal reflow (N = 152)	No reflow (N = 46)	*P* value
Age, years	56.8 ± 7.9	53.3 ± 6.1	0.693
Male, n (%)	121 (79.6)	38 (82.6)	0.390
Diabetes, n (%)	43 (28.3)	10 (21.9)	0.630
Hyperlipidemia, n (%)	13 (8.6)	6 (13.0)	0.394
Family history, n (%)	31 (20.4)	11 (23.9)	0.616
Smoking, n (%)	43 (28.3)	15 (32.6)	0.649
Chest pain to hospital time, hours	3.2 ± 1.2	3.5 ± 1.9	0.400
Door to balloon time, minutes	43.5 ± 15.3	48.6 ± 21.5	0.397

**Table 2:** Angiography and procedural features of STEMI patients.

Characteristics	Normal reflow (N = 152)	No reflow (N = 46)	*P* value
**Infarct related artery (%)**			0.603
Left anterior descending	92 (60.5)	30 (65.2)	
Left main	5 (3.3)	1 (2.2)	
Left cirumflex	32 (21.1)	9 (19.6)	
**Right coronary artery**	23 (15.1)	6 (13.0)	
**Multi-vessel disease (%)**	83 (54.6)	21 (45.7)	0.187
**Stent implantation (%)**	131 (86.2)	42 (91.3)	0.922
**Total stent length**	24.8 ± 8.3	26.5 ± 6.5	0.435

**Table 3:** Logistic regression analysis of reflow-related features of STEMI patients.

Variable	OR	95% CI	*p*-value
Age	1.189	0.984–1.348	0.452
Male	1.254	0.998–1.487	0.533
Diabetes	1.268	0.745–1.984	0.685
Hyperlipidemia	1.448	0.887–1.784	0.931
Family history	1.259	0.992–1.475	0.824
Smoking	0.968	0.789–1.325	0.361
Chest pain to hospital time	0.913	0.715–1.442	0.420
Door to balloon time	1.373	0.887–1.584	0.778
Infarct related artery	1.353	0.654–1.645	0.560
Multi-vessel disease	1.224	0.485–1.468	0.624
Stent implantation	1.461	0.558–1.874	0.973
Total stent length	1.121	0.879–1.415	0.966
MALAT1	1.356	0.336–0.848	< 0.05
miR-30e	1.162	1.298–1.298	< 0.05
miR-126	1.511	1.558–1.742	< 0.05
miR-155	1.214	0.781–1.447	0.236
CRP	1.320	1.168–1.468	< 0.05
HPSE	1.169	1.092–1.469	< 0.05
EDN1	1.413	1.148–1.687	< 0.05

### Differential expression of MALAT1, miR-30e, miR-126, miR-155, CRP, HPSE and EDN1 in the plasma of STEMI patients receiving pPCI

**Figure 1:**
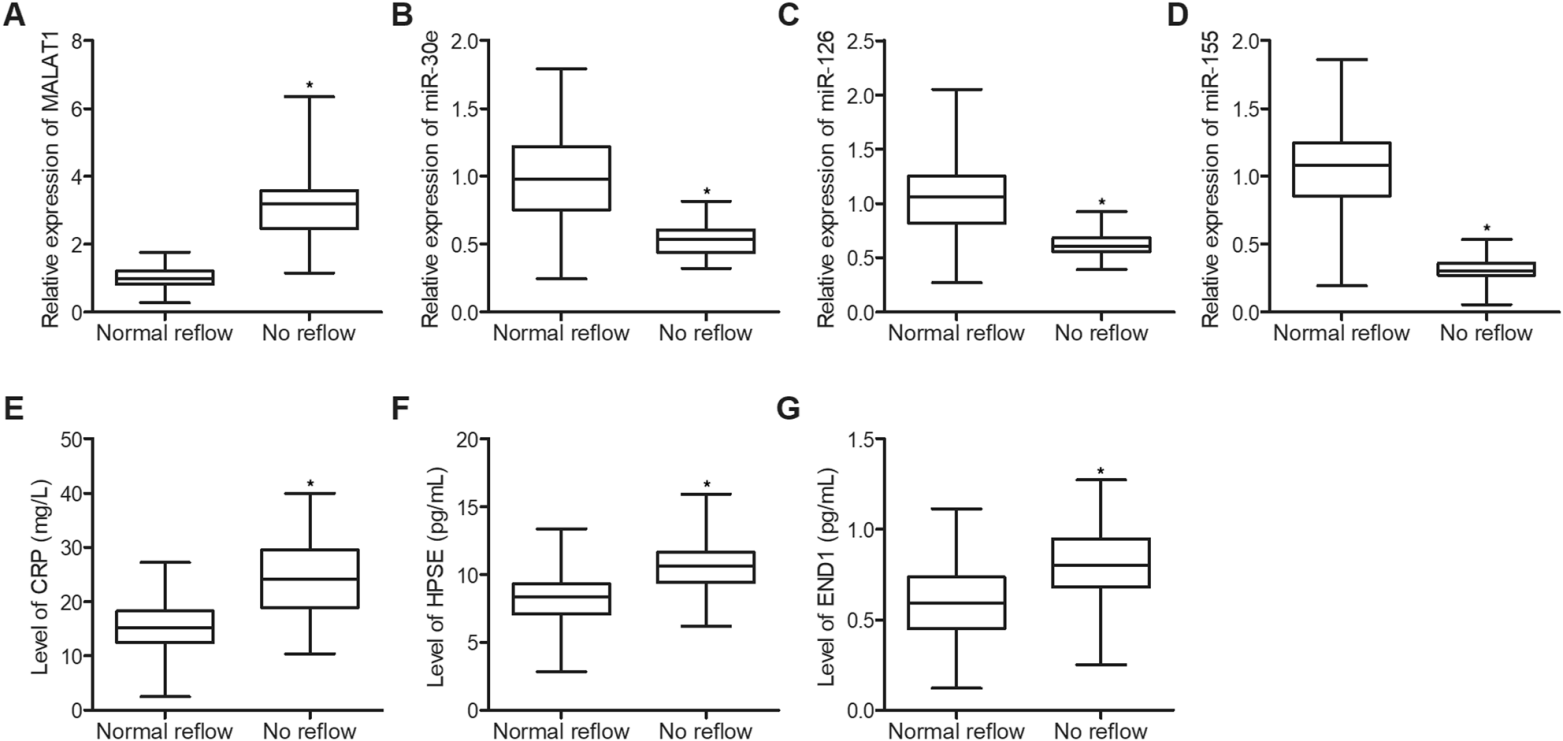
Differential expression of MALAT1, miR-30e, miR-126, miR-155, CRP, HPSE and EDN1 in the plasma of STEMI patients receiving pPCI (*P value < 0.05 vs. normal reflow group). (**A**) The expression of MALAT1 was enhanced in the plasma of STEMI patients receiving pPCI who were diagnosed with no-reflow phenomenon when compared with patients with normal reflow. (**B**) The expression of miR-30e was suppressed in the plasma of STEMI patients receiving pPCI who were diagnosed with no-reflow phenomenon when compared with patients with normal reflow. (**C**) The expression of miR-126 was suppressed in the plasma of STEMI patients receiving pPCI who were diagnosed with no-reflow phenomenon when compared with patients with normal reflow. (**D**) The expression of miR-155 was suppressed in the plasma of STEMI patients receiving pPCI who were diagnosed with no-reflow phenomenon when compared with patients with normal reflow. (**E**) The expression of CRP was increased in the plasma of STEMI patients receiving pPCI who were diagnosed with no-reflow phenomenon when compared with patients with normal reflow. (**F**) The expression of HPSE was increased in the plasma of STEMI patients receiving pPCI who were diagnosed with no-reflow phenomenon when compared with patients with normal reflow. (**G**) The expression of EDN1 was increased in the plasma of STEMI patients receiving pPCI who were diagnosed with no-reflow phenomenon when compared with patients with normal reflow.

The expression of MALAT1 was remarkably enhanced in the plasma of STEMI patients receiving pPCI who were diagnosed with no-reflow phenomenon (Fig. 1A). However, the expression of miR-30e (Fig. 1B), miR-126 (Fig. 1C) and miR-155 (Fig. 1D) was notably suppressed in the plasma of no-reflow group. Moreover, the abundance of CRP (Fig. 1E), HPSE (Fig. 1F) and EDN1 (Fig. 1G) was significantly increased in the plasma of STEMI patients receiving pPCI who were diagnosed with no-reflow phenomenon.

### The expression of MALAT1, miR-30e, miR-126 and CRP could be used as the predictive biomarker to differentiate normal reflow and no-reflow in STEMI patients receiving pPCI

**Figure 2:**
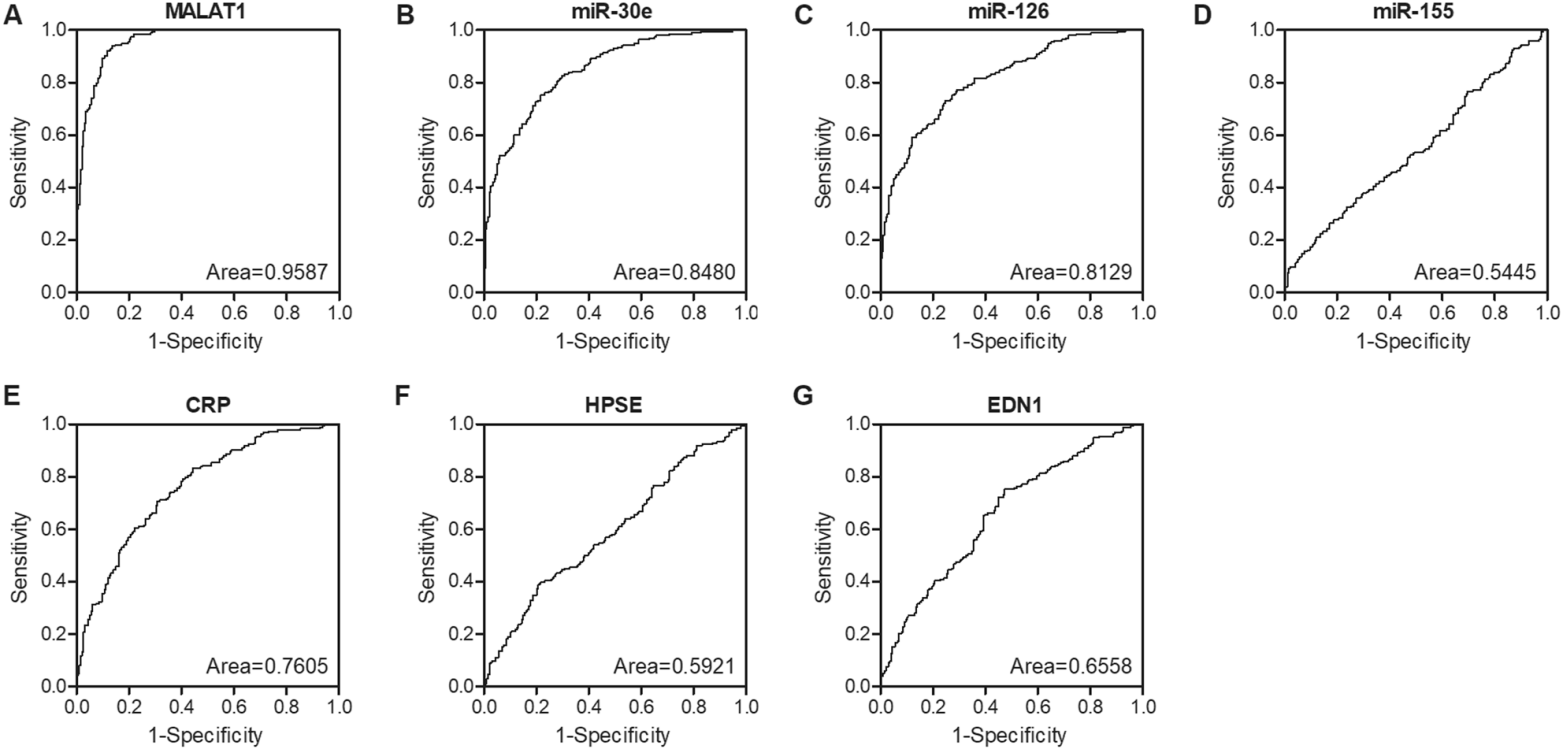
ROC analysis showed considerable efficiency of MALAT1, miR-30e, miR-126 and CRP to differentiate normal reflow and no-reflow in STEMI patients receiving pPCI. (**A**) ROC analysis of MALAT1 expression in differentiating normal reflow and no-reflow in STEMI patients receiving pPCI (95% confidence interval: 0.94 to 0.98; P value < 0.0001). (**B**) ROC analysis of miR-30e expression in differentiating normal reflow and no-reflow in STEMI patients receiving pPCI (95% confidence interval: 0.81 to 0.88; P value < 0.0001). (**C**) ROC analysis of miR-126 expression in differentiating normal reflow and no-reflow in STEMI patients receiving PCI (95% confidence interval: 0.77 to 0.85; P value < 0.0001). (**D**) ROC analysis of miR-155 expression in differentiating normal reflow and no-reflow in STEMI patients receiving pPCI (95% confidence interval: 0.49 to 0.60; P value = 0.1257). (**E**) ROC analysis of CRP expression in differentiating normal reflow and no-reflow in STEMI patients receiving pPCI (95% confidence interval: 0.81 to 0.80; P value < 0.0001). (**F**) ROC analysis of HPSE expression in differentiating normal reflow and no-reflow in STEMI patients receiving pPCI (95% confidence interval: 0.54 to 0.65; P value = 001,525). (**G**) ROC analysis of EDN1 expression in differentiating normal reflow and no-reflow in STEMI patients receiving pPCI (95% confidence interval: 0.60 to 0.71; P value < 0.0001).

Furthermore, the AUC for MALAT1 was as high as 0.95, indicating that MALAT1 was an ideal biomarker for the prediction of reflow phenomenon (Fig. 2A). Besides, the AUCs of miR-30e (Fig. 2B), miR-126 (Fig. 2C) and miR-155 (Fig. 2D) were 0.85, 0.80 and 0.55, respectively, while the AUCs of CRP (Fig. 2E), HPSE (Fig. 2F) and EDN1 (Fig. 2G) were 0.75, 0.60 and 0.65, respectively. These results indicated that the expression of MALAT1, miR-30e, miR-126 and CRP could be used as the biomarker to differentiate normal reflow and no-reflow in STEMI patients receiving pPCI.

### The luciferase activities of MALAT1 were suppressed by miR-30e, miR-126 and miR-155. The luciferase activity of HPSE was inhibited by miR-126 and the luciferase activity of EDN1 was repressed by miR-155

**Figure 3:**
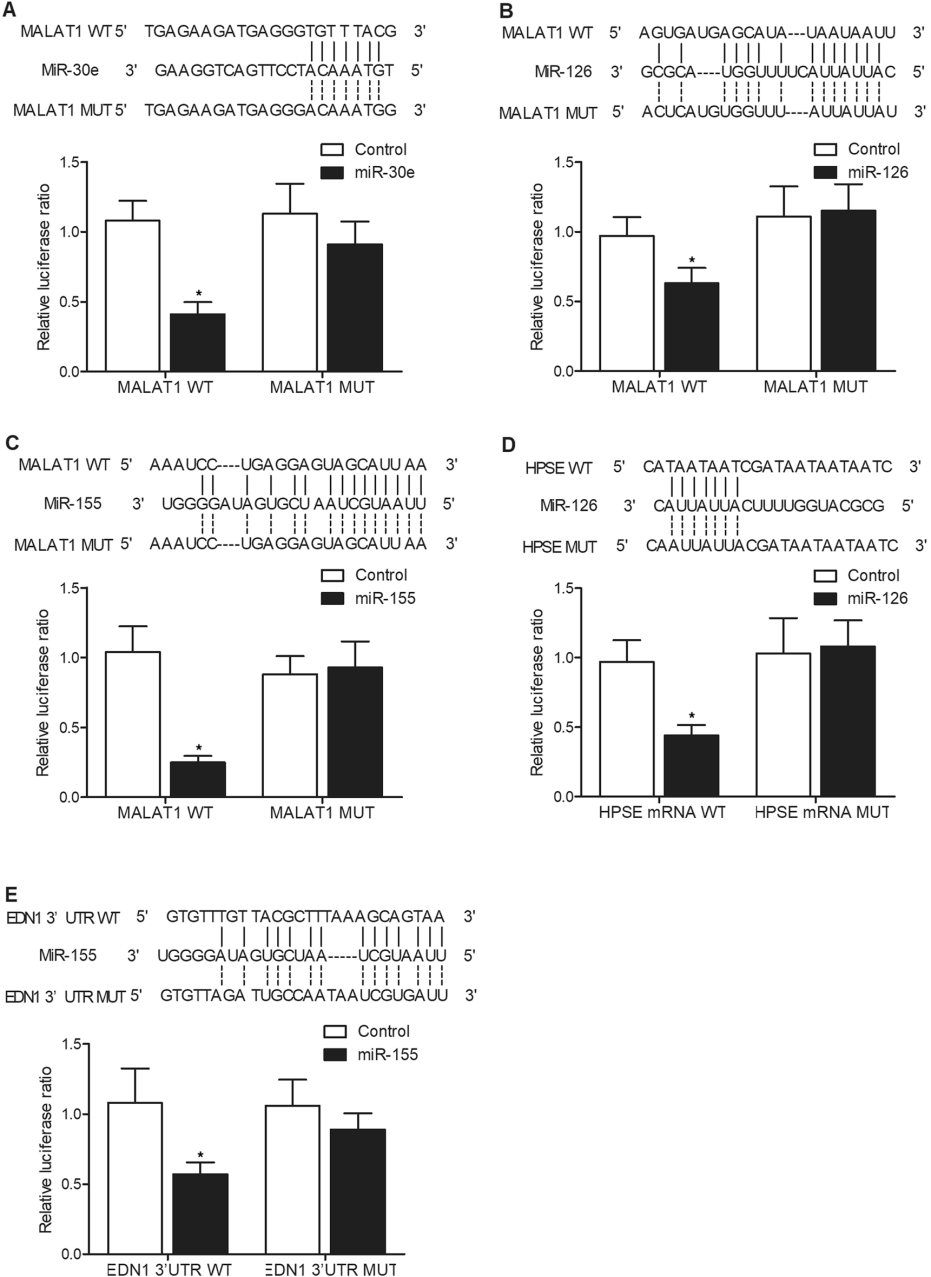
Luciferase assays were used to explore the relationship of MALAT1/miR-30e, MALAT1/miR-126, MALAT1/miR-155, miR-126/HPSE, and miR-155/EDN1 (* P value < 0.05 vs. Control group). (**A**) The luciferase activity of MALAT1 was suppressed by miR-30e in HUVEC and HAEC cells. (**B**) The luciferase activity of MALAT1 was suppressed by miR-126 in HUVEC and HAEC cells. (**C**) The luciferase activity of MALAT1 was suppressed by miR-155 in HUVEC and HAEC cells. (**D**) The luciferase activity of HPSE was suppressed by miR-126 in HUVEC and HAEC cells. (**E**) The luciferase activity of EDN1 was suppressed by miR-155 in HUVEC and HAEC cells.

Binding site screening respectively identified potential binding site of miR-30e (Fig. 3A), miR-126 (Fig. 3B) and miR-155 (Fig. 3C) in MALAT1, and the luciferase activity of wild type MALAT1 was remarkably suppressed by the respective transfection of miR-30e (Fig. 3A), miR-126 (Fig. 3B) and miR-155 (Fig. 3C) in HUVEC and HAEC cells. Meanwhile, potential binding site of miR-126 was found in the 3′ UTR of HPSE, and luciferase activity of wild type HPSE was remarkably suppressed by miR-126 in HUVEC and HAEC cells (Fig. 3D). Also, miR-155 was found to potentially bind to EDN1, and the luciferase activity of wild type EDN1 was remarkably suppressed by miR-155 in HUVEC and HAEC cells (Fig. 3E).

### MALAT1 siRNA activated the expression of miR-30e, miR-126 and miR-155, but suppressed the expression of CRP, HPSE and EDN1

**Figure 4:**
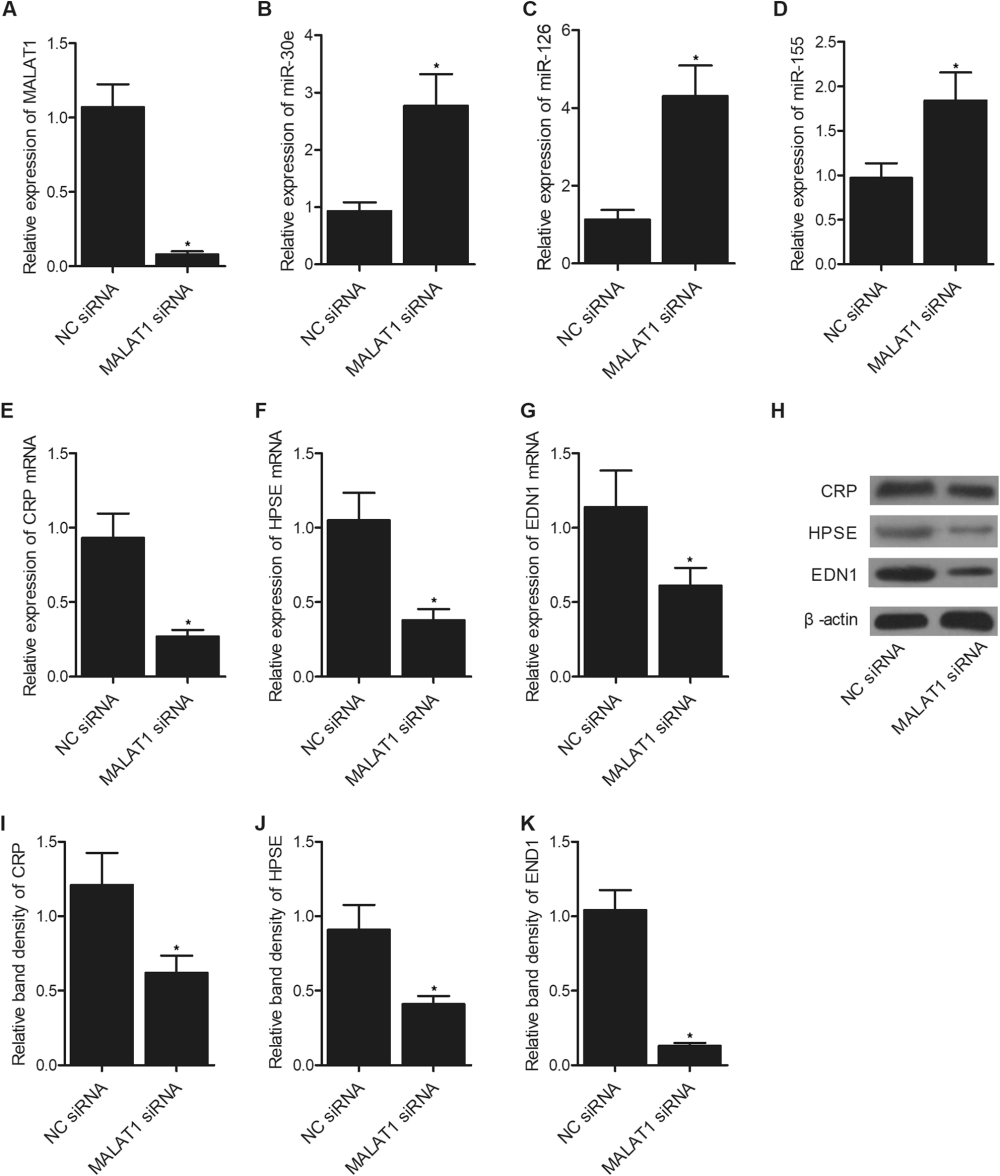
MALAT1 siRNA altered the expression of miR-30e, miR-126, miR-155, CRP, HPSE and EDN1 in HUVEC and HAEC cells (*P value < 0.05 vs. NC siRNA). (**A**) MALAT1 siRNA dramatically suppressed the expression of MALAT1 in HUVEC and HAEC cells. (**B**) MALAT1 siRNA notably enhanced the expression of miR-30e in HUVEC and HAEC cells. (**C**) MALAT1 siRNA notably enhanced the expression of miR-126 in HUVEC and HAEC cells. (**D**) MALAT1 siRNA notably enhanced the expression of miR-155 in HUVEC and HAEC cells. (**E**) MALAT1 siRNA remarkably suppressed the expression of CRP mRNA in HUVEC and HAEC cells. (**F**) MALAT1 siRNA remarkably suppressed the expression of HPSE mRNA in HUVEC and HAEC cells. (**G**) MALAT1 siRNA remarkably suppressed the expression of EDN1 mRNA in HUVEC and HAEC cells. (**H**) Western blot analysis showed that the protein expression of CRP, HPSE and EDN1 was suppressed by MALAT1 siRNA in HUVEC and HAEC cells. (**I**) Quantitative analysis showed that the protein expression of CRP was suppressed by MALAT1 siRNA in HUVEC and HAEC cells. (**J**) Quantitative analysis showed that the protein expression of HPSE was suppressed by MALAT1 siRNA in HUVEC and HAEC cells. (**K**) Quantitative analysis showed that the protein expression of EDN1 was suppressed by MALAT1 siRNA in HUVEC and HAEC cells.

The expression of MALAT1 was dramatically suppressed by MALAT1 siRNA in HUVEC and HAEC cells (Fig. 4A), but the expression of miR-30e (Fig. 4B), miR-126 (Fig. 4C) and miR-155 (Fig. 4D) was remarkably activated by MALAT1 siRNA in HUVEC and HAEC cells. On the contrary, the expression of CRP (Fig. 4E), HPSE (Fig. 4F) and EDN1 (Fig. 4G) mRNA was notably inhibited by MALAT1 siRNA in HUVEC and HAEC cells. Furthermore, the protein expression of CRP, HPSE and EDN1 was notably inhibited by MALAT1 siRNA in HUVEC and HAEC cells (Fig. 4H–K). The original blots of CRP, HPSE and EDN1 are present in the Supplementary Information.

## Discussion

In this study, we recruited 198 ST-segment elevation myocardial infarction (STEMI) patients receiving primary percutaneous coronary intervention (pPCI) and divided them into two groups according to their reflow status. QPCR was performed to evaluate the expression of MALAT1, miR-30e, miR-126, and miR-155 in the plasma of patients with normal reflow and no-reflow. The expression of MALAT1 was remarkably enhanced, while the expression of miR-30e, miR-126 and miR-155 was significantly suppressed in the plasma of STEMI patients receiving pPCI who were diagnosed with no-reflow phenomenon. In addition, ELISA was performed to analyze the expression of CRP, HPSE and EDN1 in the plasma of patients with normal reflow and no-reflow. The expression of CRP, HPSE and EDN1 was notably upregulated in the plasma of STEMI patients receiving pPCI who were diagnosed with no-reflow phenomenon.

MALAT1 was confirmed to be associated with numerous forms of tumors. For instance, MALAT1 is over-expressed in many cancer cells to control their proliferation, invasion, migration, and apoptosis^26^. Latest research also showed that lncRNA MALAT1 enhanced the invasion and proliferation of pancreatic, ovarian and glioma cancer cells by activating autophagy^27–29^. A previous research presented that MALAT1 regulated ATG5 expression and autophagy through miR-30e^30^. Likewise, it was additionally discovered that miR-30e may be inhibited by MALAT1 to up-regulate ATG5 as well as activate autophagy in GC. Furthermore, MALAT1 contains two miR-126-5p miRNA response elements (MREs). Dual luciferase, biotin-RNA pull-down, as well as RNA-IP assays all presented that MALAT1 can sponge miR-126-5p^23^. MiR-155 was first identified to play an immune regulatory role in adaptive and innate immune responses^31,32^. It was also suggested that miR-155 plays an essential role in the onset of fibrosis^33,34^. Over-expressed in fibrotic tissues, miR-155 is essential to collagen metabolism by mediating TGF-1 signaling^35–37^. In this study, we used luciferase assay to explore the regulatory relationship of MALAT1/miR-30e, MALAT1/miR-126, MALAT1/miR-155, miR-126/HPSE, and miR-155/EDN1. The luciferase activity of MALAT1 was effectively suppressed by miR-30e, miR-126 and miR-155. The luciferase activity of HPSE was inhibited by miR-126. The luciferase activity of EDN1 was repressed by miR-155. Moreover, we suppressed the expression of MALAT1 using MALAT1 siRNA. MALAT1 siRNA up-regulated the expression of miR-30e, miR-126 and miR-155, but suppressed the expression of CRP, HPSE and EDN1.

It was show that pre-PCI plasma miRNA-30e expression in STEMI patients could be used to identify the risk of no-reflow. Furthermore, it was shown that the plasma levels of miRNA-30e were positively and significantly correlated with the level of LVEF upon admission, while negatively and considerably correlated with the expression level of hs-CRP. As a result of the rapid rise of CRP level in inflammatory conditions, CRP is considered as an important protein involved in the acute phase of inflammation by playing a significant role in mental stress, anxiety, neoplastic disease and myocardial infarction^2,38,39^.

Indicators like endothelin as well as CRP play an important regulatory role during the progression of no-reflow^40^. In a recent study, the hs-CRP and ET-1 levels in the post-PCI peripheral blood were significantly elevated in the no-reflow group (P < 0.05). Moreover, the serum levels of hs-CRP showed the largest reduction within 3 h in the reflow group after PCI^41^. A number of mechanisms were proposed to support the hypothesis that high ET-1 levels may be used as a potential biomarker for the prediction of no-reflow phenomenon. As one of strongest vasoconstrictors in the body, ET-1 is produced and secreted from the vascular endothelia upon injury^42^. ET-1 also targets coronary arteries with low resistance. It has been revealed that the level of ET-1 was elevated in patients with ischemia ailments^43^. In patients with renal ischemia/reperfusion injury, the deletion of ET-1 from endothelial tissues protects the normal function of kidneys^44^. Moreover, ET-1 can additionally interact with polymorphonuclear leukocytes to induce no-reflow phenomenon by boosting the adherence of PMN leukocytes to endothelial tissues, therefore promoting the plugging of PMN^45^. Finally, ET-1 may enhance microvascular compression by elevating the permeability of microvasculature and by inducing edema^46^. Consequently, it is likely that the secretion of ET-1 in endothelia injured by ischemia may trigger sustained and rigorous microvascular constriction to potentiate no-reflow.

Heparanase is ubiquitously expressed to regulate angiogenesis, tumorigenesis, autophagy, fibrosis, and inflammation by cleaving HS groups from proteoglycans. The HS removal then lowers the stability of endothelial cell barriers while regulating the communication with cytokines, growth factors and chemokines^47^. The logistic regression revealed that miR-126 and also hs-CRP are independent risk factors of coronary slow flow. In addition, the plasma expression of miR-126 was significantly associated with the level of CSF, thus miR-126 may be utilized as a valuable predictor of coronary slow flow^48^. Elevated level of HPA in STEMI patients was associated with a high level of TB. Moreover, elevated level of HPA may predict thrombotic issues like no-reflow in STEMI patients^17^. In this study, we performed ROC analysis to evaluate the diagnostic potential of MALAT1, miR-30e, miR-126, miR-155, CRP, HPSE and EDN1 in differentiating normal reflow and no-reflow in STEMI patients receiving pPCI. MALAT1, miR-30e, miR-126 and CRP showed considerable efficiency in differentiating normal reflow and no-reflow in STEMI patients receiving pPCI.

The computational analysis indicated the MALAT1 as a regulator of miR-30e/CRP, miR-126/HPSE, and miR-155/END1. Furthermore, luciferase reporter assay results showed that there are interactions between MALAT1 and those three miRNAs (miR-30e, miR-126, and miR-155) and the CRP, HPSE, and END1 are the direct genes of miR-30e, miR-126, and miR-155, respectively. Those results were also confirmed by the results of cellular transfection of miRNA mimics. All those results that MALAT1 is a upstream regulator, which might be the basis for its role as a better biomarker for no-reflow phenomenon.

## Conclusion

In this study, we demonstrated that MALAT1 could respectively sponge the expression of miR-30e, miR-126 and miR-155. The subsequent assays validated that miR-30e, miR-126 and miR-155 targeted CRP, HPSE and EDN1 in a negative manner. Therefore, since MALAT1 was found to present the highest AUC value among all studied genes, MALAT1 was shown to function as a biomarker of no-reflow phenomenon in STEMI patients who were subjected to pPCI.

## Supplementary Information

Supplementary Information.

## Data Availability

The data of this study are available from the corresponding author upon reasonable request.

## Abbreviations

HUVECs: Human umbilical vein endothelial cells
HAECs: Human aortic endothelial cells
STEMI: ST-segment elevated myocardial infarction
PCI: Percutaneous coronary intervention
HPSE: Heparanase
EDN-1: Endothelin

## References

[1] Jaffe R, Charron T, Puley G, Dick A, Strauss BH (2008). Microvascular obstruction and the no-reflow phenomenon after percutaneous coronary intervention. Circulation.

[2] Durante A, Camici PG (2015). Novel insights into an "old" phenomenon: The no reflow. Int. J. Cardiol..

[3] Harrison RW, Aggarwal A, Ou FS, Klein LW, Rumsfeld JS, Roe MT, Wang TY (2013). American College of Cardiology National Cardiovascular Data R: Incidence and outcomes of no-reflow phenomenon during percutaneous coronary intervention among patients with acute myocardial infarction. Am. J. Cardiol..

[4] Ndrepepa G, Tiroch K, Fusaro M, Keta D, Seyfarth M, Byrne RA, Pache J, Alger P, Mehilli J, Schomig A, Kastrati A (2010). 5-year prognostic value of no-reflow phenomenon after percutaneous coronary intervention in patients with acute myocardial infarction. J. Am. Coll. Cardiol..

[5] Ipek G, Onuk T, Karatas MB, Gungor B, Osken A, Keskin M, Oz A, Tanik O, Hayiroglu MI, Yaka HY, Ozturk R, Bolca O (2016). CHA2DS2-VASc score is a predictor of no-reflow in patients with ST-segment elevation myocardial infarction who underwent primary percutaneous intervention. Angiology.

[6] Dahariya S, Paddibhatla I, Kumar S, Raghuwanshi S, Pallepati A, Gutti RK (2019). Long non-coding RNA: Classification, biogenesis and functions in blood cells. Mol. Immunol..

[7] Fatica A, Bozzoni I (2014). Long non-coding RNAs: New players in cell differentiation and development. Nat. Rev. Genet..

[8] Ji P, Diederichs S, Wang W, Boing S, Metzger R, Schneider PM, Tidow N, Brandt B, Buerger H, Bulk E, Thomas M, Berdel WE, Serve H, Muller-Tidow C (2003). MALAT-1, a novel noncoding RNA, and thymosin beta4 predict metastasis and survival in early-stage non-small cell lung cancer. Oncogene.

[9] Sun W, Yang Y, Xu C, Guo J (2017). Regulatory mechanisms of long noncoding RNAs on gene expression in cancers. Cancer Genet..

[10] Zhang X, Hamblin MH, Yin KJ (2017). The long noncoding RNA Malat 1: Its physiological and pathophysiological functions. RNA Biol..

[11] Wiese D, Kampe K, Waldmann J, Heverhagen AE, Bartsch DK, Fendrich V (2016). C-reactive protein as a new prognostic factor for survival in patients with pancreatic neuroendocrine neoplasia. J. Clin. Endocrinol. Metab..

[12] Adderley SR, Fitzgerald DJ (1999). Oxidative damage of cardiomyocytes is limited by extracellular regulated kinases 1/2-mediated induction of cyclooxygenase-2. J. Biol. Chem..

[13] Kuwano T, Nakao S, Yamamoto H, Tsuneyoshi M, Yamamoto T, Kuwano M, Ono M (2004). Cyclooxygenase 2 is a key enzyme for inflammatory cytokine-induced angiogenesis. FASEB J..

[14] Samad TA, Moore KA, Sapirstein A, Billet S, Allchorne A, Poole S, Bonventre JV, Woolf CJ (2001). Interleukin-1beta-mediated induction of Cox-2 in the CNS contributes to inflammatory pain hypersensitivity. Nature.

[15] Caruana I, Savoldo B, Hoyos V, Weber G, Liu H, Kim ES, Ittmann MM, Marchetti D, Dotti G (2015). Heparanase promotes tumor infiltration and antitumor activity of CAR-redirected T lymphocytes. Nat. Med..

[16] Tran VM, Wade A, McKinney A, Chen K, Lindberg OR, Engler JR, Persson AI, Phillips JJ (2017). Heparan sulfate glycosaminoglycans in glioblastoma promote tumor invasion. Mol. Cancer Res..

[17] Gurbuz AS, Ozturk S, Efe SC, Yilmaz MF, Yanik RE, Yaman A, Kirma C (2019). Heparanase is a predictive marker for high thrombus burden in patients with ST-segment elevation myocardial infarction. Biomarkers.

[18] Mayyas F, Al-Jarrah M, Ibrahim K, Mfady D, Van Wagoner DR (2015). The significance of circulating endothelin-1 as a predictor of coronary artery disease status and clinical outcomes following coronary artery catheterization. Cardiovasc. Pathol..

[19] Guddeti RR, Prasad A, Matsuzawa Y, Aoki T, Rihal C, Holmes D, Best P, Lennon RJ, Lerman LO, Lerman A (2016). Role of endothelin in microvascular dysfunction following percutaneous coronary intervention for non-ST elevation acute coronary syndromes: A single-centre randomised controlled trial. Open Heart.

[20] Acet H, Ertas F, Akil MA, Bilik MZ, Aydin M, Polat N, Yildiz A (2016). The utility of the TIMI risk index on admission for predicting angiographic no-reflow after primary percutaneous coronary intervention in patients with STEMI. Turk. J. Med. Sci..

[21] Wang Q, Lu G, Chen Z (2019). MALAT1 promoted cell proliferation and migration via MALAT1/miR-155/MEF2A pathway in hypoxia of cardiac stem cells. J. Cell Biochem..

[22] Zhang YF, Li CS, Zhou Y, Lu XH (2020). Propofol facilitates cisplatin sensitivity via lncRNA MALAT1/miR-30e/ATG5 axis through suppressing autophagy in gastric cancer. Life Sci..

[23] Sun Z, Ou C, Liu J, Chen C, Zhou Q, Yang S, Li G, Wang G, Song J, Li Z, Zhang Z, Yuan W, Li X (2019). YAP1-induced MALAT1 promotes epithelial-mesenchymal transition and angiogenesis by sponging miR-126-5p in colorectal cancer. Oncogene.

[24] Su Q, Ye Z, Sun Y, Yang H, Li L (2018). Relationship between circulating miRNA-30e and no-reflow phenomenon in STEMI patients undergoing primary coronary intervention. Scand. J. Clin. Lab. Invest..

[25] Lewicki L, Siebert J, Marek-Trzonkowska N, Masiewicz E, Kolinski T, Reiwer-Gostomska M, Targonski R, Trzonkowski P (2015). Elevated serum tryptase and endothelin in patients with ST segment elevation myocardial infarction: preliminary report. Mediators Inflamm..

[26] Zhao M, Wang S, Li Q, Ji Q, Guo P, Liu X (2018). MALAT1: A long non-coding RNA highly associated with human cancers. Oncol Lett..

[27] Li L, Chen H, Gao Y, Wang YW, Zhang GQ, Pan SH, Ji L, Kong R, Wang G, Jia YH, Bai XW, Sun B (2016). Long noncoding RNA MALAT1 promotes aggressive pancreatic cancer proliferation and metastasis via the stimulation of autophagy. Mol. Cancer Ther..

[28] Fu Z, Luo W, Wang J, Peng T, Sun G, Shi J, Li Z, Zhang B (2017). Malat1 activates autophagy and promotes cell proliferation by sponging miR-101 and upregulating STMN1, RAB5A and ATG4D expression in glioma. Biochem. Biophys. Res. Commun..

[29] Hu J, Zhang L, Mei Z, Jiang Y, Yi Y, Liu L, Meng Y, Zhou L, Zeng J, Wu H, Jiang X (2018). Interaction of E3 ubiquitin ligase MARCH7 with long noncoding RNA MALAT1 and autophagy-related protein ATG7 promotes autophagy and invasion in ovarian cancer. Cell Physiol. Biochem..

[30] Ye Y, Fang Y, Xu W, Wang Q, Zhou J, Lu R (2016). 3,3'-Diindolylmethane induces anti-human gastric cancer cells by the miR-30e-ATG5 modulating autophagy. Biochem. Pharmacol..

[31] Vigorito E, Perks KL, Abreu-Goodger C, Bunting S, Xiang Z, Kohlhaas S, Das PP, Miska EA, Rodriguez A, Bradley A, Smith KG, Rada C, Enright AJ, Toellner KM, Maclennan IC, Turner M (2007). microRNA-155 regulates the generation of immunoglobulin class-switched plasma cells. Immunity.

[32] Thai TH, Calado DP, Casola S, Ansel KM, Xiao C, Xue Y, Murphy A, Frendewey D, Valenzuela D, Kutok JL, Schmidt-Supprian M, Rajewsky N, Yancopoulos G, Rao A, Rajewsky K (2007). Regulation of the germinal center response by microRNA-155. Science.

[33] Artlett CM, Sassi-Gaha S, Hope JL, Feghali-Bostwick CA, Katsikis PD (2017). Mir-155 is overexpressed in systemic sclerosis fibroblasts and is required for NLRP3 inflammasome-mediated collagen synthesis during fibrosis. Arthritis Res. Ther..

[34] Pottier N, Maurin T, Chevalier B, Puissegur MP, Lebrigand K, Robbe-Sermesant K, Bertero T, Lino Cardenas CL, Courcot E, Rios G, Fourre S, Lo-Guidice JM, Marcet B, Cardinaud B, Barbry P, Mari B (2009). Identification of keratinocyte growth factor as a target of microRNA-155 in lung fibroblasts: implication in epithelial-mesenchymal interactions. PLoS ONE.

[35] Liu L, Liu X, Ren X, Tian Y, Chen Z, Xu X, Du Y, Jiang C, Fang Y, Liu Z, Fan B, Zhang Q, Jin G, Yang X, Zhang X (2016). Smad2 and Smad3 have differential sensitivity in relaying TGFbeta signaling and inversely regulate early lineage specification. Sci. Rep..

[36] Chen W, Lam SS, Srinath H, Schiffer CA, Royer WE, Lin K (2007). Competition between Ski and CREB-binding protein for binding to Smad proteins in transforming growth factor-beta signaling. J. Biol. Chem..

[37] Wang J, He W, Xu X, Guo L, Zhang Y, Han S, Shen D (2017). The mechanism of TGF-beta/miR-155/c-Ski regulates endothelial-mesenchymal transition in human coronary artery endothelial cells. Biosci. Rep..

[38] Brosh D, Assali AR, Mager A, Porter A, Hasdai D, Teplitsky I, Rechavia E, Fuchs S, Battler A, Kornowski R (2007). Effect of no-reflow during primary percutaneous coronary intervention for acute myocardial infarction on six-month mortality. Am. J. Cardiol..

[39] Celik T, Balta S, Mikhailidis DP, Ozturk C, Aydin I, Tok D, Yildirim AO, Demir M, Iyisoy A (2017). The relation between no-reflow phenomenon and complete blood count parameters. Angiology.

[40] Song R, Chou YI, Kong J, Li J, Pan B, Cui M, Zhou E, Zhang Y, Zheng L (2015). Association of endothelial microparticle with NO, eNOS, ET-1, and fractional flow reserve in patients with coronary intermediate lesions. Biomarkers.

[41] Liu M, Liang T, Zhang P, Zhang Q, Lu L, Wang Z (2017). hsCRP and ET-1 expressions in patients with no-reflow phenomenon after Percutaneous Coronary Intervention. Pak. J. Med. Sci..

[42] Bourque SL, Davidge ST, Adams MA (2011). The interaction between endothelin-1 and nitric oxide in the vasculature: New perspectives. Am. J. Physiol. Regul. Integr. Comp. Physiol..

[43] Ziv I, Fleminger G, Djaldetti R, Achiron A, Melamed E, Sokolovsky M (1992). Increased plasma endothelin-1 in acute ischemic stroke. Stroke.

[44] Arfian N, Emoto N, Vignon-Zellweger N, Nakayama K, Yagi K, Hirata K (2012). ET-1 deletion from endothelial cells protects the kidney during the extension phase of ischemia/reperfusion injury. Biochem. Biophys. Res. Commun..

[45] Sato Y, Hogg JC, English D, van Eeden SF (2000). Endothelin-1 changes polymorphonuclear leukocytes' deformability and CD11b expression and promotes their retention in the lung. Am. J. Respir. Cell Mol. Biol..

[46] del Zoppo GJ, Hallenbeck JM (2000). Advances in the vascular pathophysiology of ischemic stroke. Thromb. Res..

[47] Changyaleket B, Deliu Z, Chignalia AZ, Feinstein DL (2017). Heparanase: Potential roles in multiple sclerosis. J. Neuroimmunol..

[48] Wang L, Wang HN (2019). Zu XL [Relationship between plasma miR-126 and coronary slow flow phenomenon]. Zhonghua Yi Xue Za Zhi.

