# Supplementary material for: LncRNA MALAT1 functions as a biomarker of no-reflow phenomenon in ST-segment elevation myocardial infarction patients receiving primary percutaneous coronary intervention
Source: Sci Rep. 2022 Feb 28;12:3294. doi: 10.1038/s41598-022-06923-z (PMC8885644; doi:10.1038/s41598-022-06923-z)

LncRNA MALAT1 functions as a biomarker of no-reflow phenomenon in ST-segment elevation myocardial infarction patients receiving primary percutaneous coronary intervention

Xiheng Yang[1*], Rixin Dai[1], Zhong Qin[1], Ruping Cai[1], Yuli Xu[1], Qiang Su[1]

1. Department of Cardiology, Affiliated Hospital of Guilin Medical University, Guilin, Guangxi, China

***Corresponding author:** Xiheng Yang

Affiliation: Department of Cardiology, Affiliated Hospital of Guilin Medical University, Guilin, Guangxi, China

Address: 15#, Lequn Road, Guilin, Guangxi 541001, China

Email: yangxiheng541001@163.com

Original WB blots

Fig 4H CRP

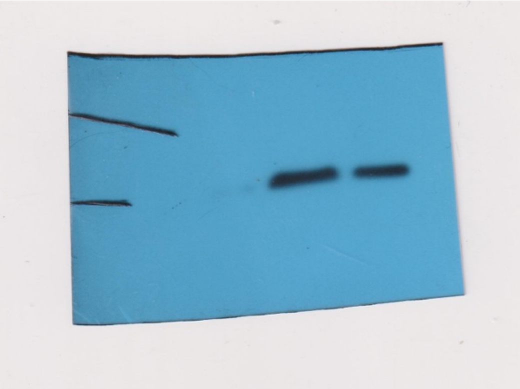

Fig 4H HPSE

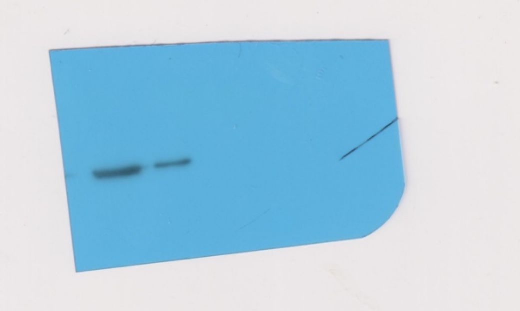

Fig 4H EDN1

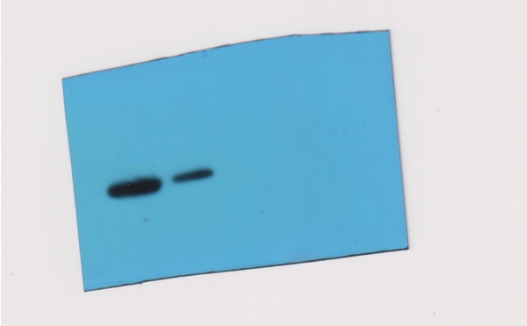

Fig 4H beta actin

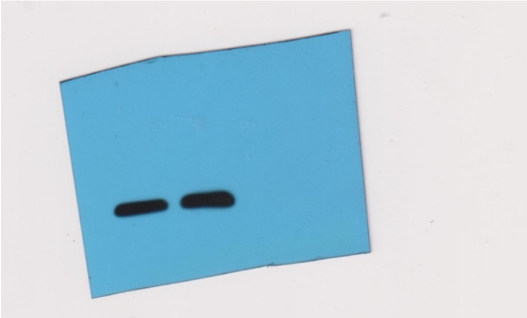

Supplement: Supplementary file 1 — Supplementary Information. [file 41598_2022_6923_MOESM1_ESM.pdf]
